# Ethnic inequalities in cancer incidence and mortality: census-linked cohort studies with 87 million years of person-time follow-up

**DOI:** 10.1186/s12885-016-2781-4

**Published:** 2016-09-26

**Authors:** Andrea M. Teng, June Atkinson, George Disney, Nick Wilson, Diana Sarfati, Melissa McLeod, Tony Blakely

**Affiliations:** Department of Public Health, University of Otago, 23a Mein Street, Newtown, Wellington, 6021 New Zealand

**Keywords:** Ethnic inequalities, Cancer mortality, Cancer incidence, Obesity, Tobacco, Infectious agents

## Abstract

**Background:**

Cancer makes up a large and increasing proportion of excess mortality for indigenous, marginalised and socioeconomically deprived populations, and much of this inequality is preventable. This study aimed to determine which cancers give rise to changing ethnic inequalities over time.

**Methods:**

New Zealand census data from 1981, 1986, 1991, 1996, 2001, and 2006, were all probabilistically linked to three to five subsequent years of mortality (68 million person-years) and cancer registrations (87 million person years) and weighted for linkage bias. Age-standardised rate differences (SRDs) for Māori (indigenous) and Pacific peoples, each compared to European/Other, were decomposed by cancer type.

**Results:**

The absolute size and percentage of the cancer contribution to excess mortality increased from 1981–86 to 2006–11 in Māori males (SRD 72.5 to 102.0 per 100,000) and females (SRD 72.2 to 109.4), and Pacific females (SRD −9.8 to 42.2) each compared to European/Other.

Specifically, excess mortality (SRDs) increased for breast cancer in Māori females (linear trend *p* < 0.01) and prostate (*p* < 0.01) and colorectal cancers (*p* < 0.01) in Māori males. The incidence gap (SRDs) increased for breast (Māori and Pacific females *p* < 0.01), endometrial (Pacific females *p* < 0.01) and liver cancers (Māori males *p* = 0.04), and for cervical cancer it decreased (Māori females *p* = 0.03). The colorectal cancer incidence gap which formerly favoured Māori, decreased for Māori males and females (*p* < 0.01).

The greatest contributors to absolute inequalities (SRDs) in mortality in 2006–11 were lung cancer (Māori males 50 %, Māori females 44 %, Pacific males 81 %), breast cancer (Māori females 18 %, Pacific females 23 %) and stomach cancers (Māori males 9 %, Pacific males 16 %, Pacific females 20 %). The top contributors to the ethnic gap in cancer incidence were lung, breast, stomach, endometrial and liver cancer.

**Conclusions:**

A transition is occurring in what diseases contribute to inequalities. The increasing excess incidence and mortality rates in several obesity- and health care access-related cancers provide a sentinel warning of the emerging drivers of ethnic inequalities. Action to further address inequalities in cancer burden needs to be multi-pronged with attention to enhanced control of tobacco, obesity, and carcinogenic infectious agents, and focus on addressing access to effective screening and quality health care.

**Electronic supplementary material:**

The online version of this article (doi:10.1186/s12885-016-2781-4) contains supplementary material, which is available to authorized users.

## Background

Indigenous, marginalised and socioeconomically deprived populations in countries around the world experience greater levels of premature mortality than their counterparts (henceforth termed excess mortality). Cancer contributes to a large and increasing proportion of this excess mortality [1, 2]. A substantial proportion of inequalities in cancer are considered to be preventable through the control of tobacco, obesity, alcohol and infectious diseases [3], with further gains likely to be realised through equal access and quality of health care [4]. Changes in risk factor prevalence and cancer detection and treatment have been associated with declines in cancer mortality rates in many countries, however not all ethnic groups have benefited equally.

Several studies document the extent of ethnic and indigenous inequalities in cancer incidence and mortality [5, 6] and explore trends in inequalities over time [7, 8]. Much of the excess cancer incidence and mortality observed in indigenous and ethnic minority groups is due to causes associated with poverty and social exclusion, particularly tobacco smoking, chronic infections, obesity and lower screening coverage [4]. However, the relative importance of these more proximal causes of cancer change over time, potentially requiring changes in the emphasis of policies aimed at addressing inequities in cancer outcomes.

**Fig. 1:**
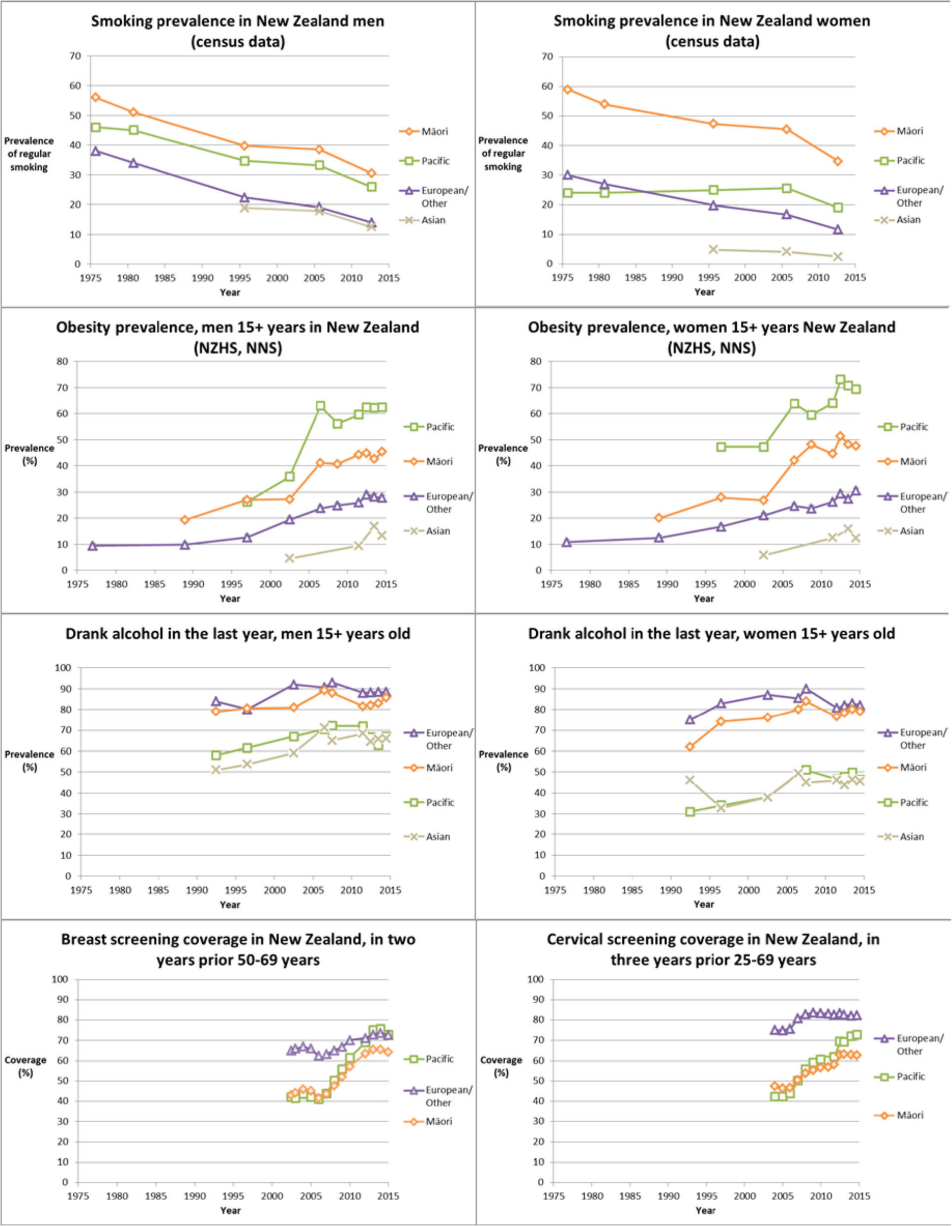
Ethnic inequalities in smoking, obesity, alcohol and screening examples of mediators for excess cancer mortality, New Zealand. Note: Smoking prevalence rates use the definition of regular current smoking of 1+ cigarette per day and there was some slight variation between censuses in the questions for ethnicity and smoking. The crude obesity prevalence rates from National Nutrition Surveys (NNS) and New Zealand Health Surveys (NZHS) use an obesity definition of a BMI of ≥30, except for Māori and Pacific peoples before 2000 when it was ≥32. The age group was 15+ year olds except for in 1977 (20–64 years) and 1989 (15–74 years). European/Other obesity figures in 1977 and 1989 are estimated from the total population. Alcohol consumption is from the NZHS and the New Zealand Drug and Alcohol Survey. Breast and cervical screening coverage is from the Independent Monitoring Reports at www.nsu.govt.nz

These issues are relevant in New Zealand, where the prevalence of tobacco smoking, obesity, human papilloma virus (HPV) infection, *H. Pylori* infection and chronic hepatitis B infection is substantially higher in Māori and Pacific peoples compared with European/Other, and screening coverage rates are lower and vary over time (Fig. 1). Such large ethnic differences in risk factors makes New Zealand a potentially valuable case study to explore the trends in ethnic inequalities in cancer burden.

A major challenge in examining ethnic inequalities in cancer and other diseases is the inconsistent collection of ethnicity data across datasets [9]. One way to address this issue is to individually link census and cancer data [8]. The linkage of census and cancer registration data in New Zealand allows a rigorous analysis providing a common measure of ethnicity across cancer datasets and census denominators that avoids (usually) underestimation of cancer rates in the indigenous population [10]. Cancer is the most common cause of mortality in New Zealand, an ethnically diverse high-income country (75 % of the New Zealand population identified as European in the 2013 census, 15 % as Māori the indigenous population, 12 % Asian and 7 % Pacific, where each individual can identify with one or more ethnic groups). Māori and Pacific peoples experience greater socioeconomic deprivation, greater cancer mortality and greater all-cause mortality than European/Other. Māori and Pacific mortality has generally declined in the 2000s [11], but inequalities compared to European/Other remain high.

Our aim was first to quantify the contribution of cancer to overall ethnic gaps in all-cause mortality over time in the New Zealand population. Second, we aimed to measure the contribution of individual cancers to the overall ethnic gaps in cancer mortality. Third, we aimed to estimate the contribution of changes in cancer incidence to trends in ethnic inequalities in mortality.

## Methods

### Data linkage

New Zealand mortality and Cancer Registry data were probabilistically linked with five-yearly censuses of population and dwellings. The population-based cancer register collects information on all malignant tumours (except basal and squamous cell skin cancers) with mandatory notification since 1994 (1993 Cancer Registry Act) and high compliance. Six closed cohorts were created of the New Zealand usual resident population (all ages) on census nights in 1981, 1986, 1991, 1996, 2001, 2006 and these populations were followed up for 5 years for incident deaths (2001–06 and 2006–11 cohorts) and 3 years for the earlier cohorts. Follow-up of incident cancer(s) was for 5 years, or in the case of the 2001 census, until 31 December 2004 (due to the timing of previous record linkage study). The probabilistic record linkage was done with QualityStage software using an individual’s address (meshblock or census area unit), sex, date of birth, ethnicity, and country of birth as matching variables. This provided 300 285 incident cancers arising from 87.3 million person-years of follow-up (1981–86 to 2006–11), and 87 606 cancer deaths from 67.9 million person-years of follow-up (1981–84 to 2006–11).

The percentage of deaths linked to a census record ranged from 71 % (1981 mortality linkage) [12] to 83 % (2006 registration linkage) [13]. Therefore, all linked census-cancer records were weighted up to be representative of all eligible cancers, using the inverse of the probability of being linked. For example, if only 20 out of 25 eligible cancers for Māori males aged 50–54 years old of high deprivation living in the north of New Zealand, were linked back to their census record, each of the 20 linked records was weighted up by 25/20 = 1.25. This adjusts for underestimation of rates using the linked datasets, and corrects for any linkage bias where the percentage of eligible cancer records linked varied by ethnicity. Further details are published elsewhere [14].

### Ethnicity and selected cancers

If individuals self-identified as Māori, Pacific and/or Asian ethnic groups, they were assigned to all the groups to which they identified; a total response ethnicity approach [15]. The remaining group that did not identify as Māori, Pacific or Asian was assigned to be European/Other, used in this study as the comparator. The 1981 census question was based on ethnic origin (rather than affiliation). To be consistent with later census years, someone was classified as Māori if they self-reported any Māori origin (likewise for Pacific and Asian). Analyses for the Asian population were limited by small numbers so they are presented in Additional file 1.

We present data on total cancer incidence and total cancer mortality. Furthermore nine cancers were selected from a wider list of 25 primary cancers. Coding was according to the International Classification of Disease (ICD 9 and 10). Stomach (C16), colorectal (C18-20), liver (C22), lung (C34), melanoma (C43), breast (C50), cervix (C53), endometrial (C54) and prostate (C61) cancers were selected because the incidence rate differences were statistically significant in at least four out of six cohorts for either sex for at least one of Māori or Pacific compared to European/Other ethnic group. Mortality rate differences were presented for all these cancers except for endometrial, cervical and liver cancer mortality where there was a smaller number of deaths. Incidence observations were censored upon the occurrence of the first relevant cancer. We could not censor for non-cancer mortality and out migration because census-cancer and census-mortality datasets were not linked in earlier cohorts. In the 2006–11 cohort, however, we did censor for non-cancer mortality.

The major risk factors for the cancers selected in our study were tobacco (lung cancer), infectious agents (stomach and liver cancer), obesity (endometrial and postmenopausal breast cancer, and to a lesser extent for colorectal cancer) and access to screening and treatment (cervical, prostate [16], and melanoma cancers) [3]. Changing reproductive patterns associated with access to contraception and later child bearing, may also be drivers of increasing breast cancer rates. Colorectal screening has been little used in New Zealand to date and is unlikely to have had much impact on incidence or mortality. Alcohol consumption is a risk factor for liver, breast and colorectal cancers [17, 18].

### Analysis

Direct age-standardisation was applied, using the WHO World Standard Population to maximise international comparability. Standardised rates were calculated in 1–74 year olds for each ethnic group in each cohort (number of events per person-years of follow-up). Standardised rate differences (SRDs) and standardised rate ratios (SRRs) were calculated for Māori and Pacific compared to European/Other. We present absolute (SRDs) and relative measures of inequality (SRRs). Absolute measures were presented in stacked diagrams because these measures are less prone to be misleading for clinical practice and public policy [19], and are more pliable to decompose absolute inequalities by cancer type. Statistical tests of increasing or decreasing linear trend (linear regression) were calculated on log rates, log rate ratios and rate differences, using the mid-date of each cohort period as the independent variable. Analysis was done using SAS V.9.4 (SAS Institute Inc, Cary, North Carolina, USA).

## Results

### Ethnic inequalities in cancer mortality

**Fig. 2:**
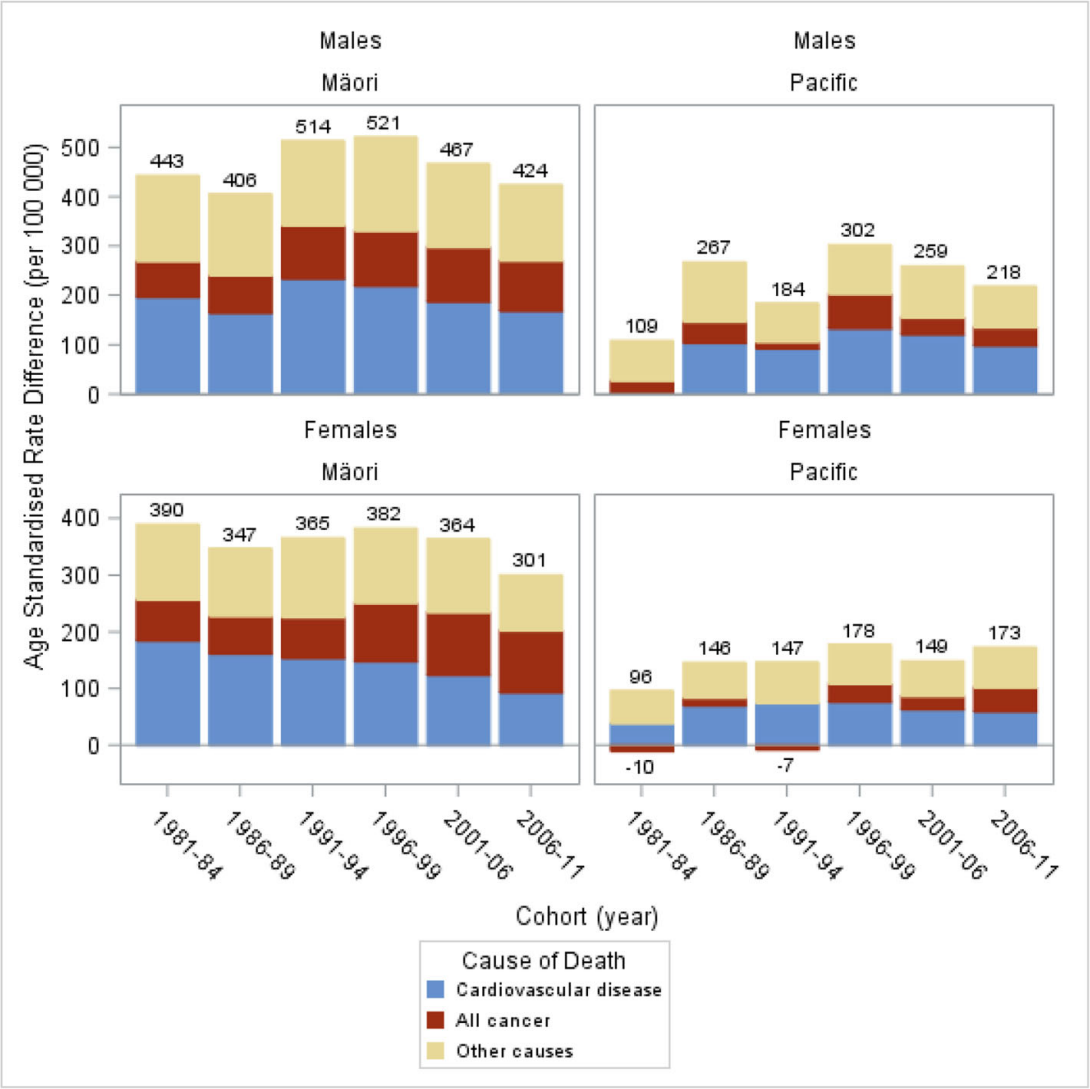
Contribution of cancer and cardiovascular disease (CVD) to ethnic inequalities in all-cause mortality over time for Māori and Pacific peoples (compared to the European/Other population) 1–74 years old in New Zealand, census-linked mortality data for six cohorts between 1981 and 2011

All-cause mortality declined for all ethnic groups across all six cohorts from 1981–84 to 2006–11. However, ethnic inequalities in all-cause mortality remained and were comprised of cardiovascular disease, cancer and other causes. The contribution of cancer to all-cause mortality inequalities increased (both in absolute and percentage terms) for Māori males and females, and Pacific females each compared to European/Other (the stacked height of ‘All cancer’ in Fig. 2). For example, inequalities (SRD) in cancer mortality between Māori and European/Other females comprised 19 % of all-cause mortality inequalities in the 1981–84 cohort (SRD 72.5/389.5 per 100 000) but increased substantially in the 2006–11 cohort (to 34 %, SRD 102.0/300.7 per 100 000).

**Fig. 3:**
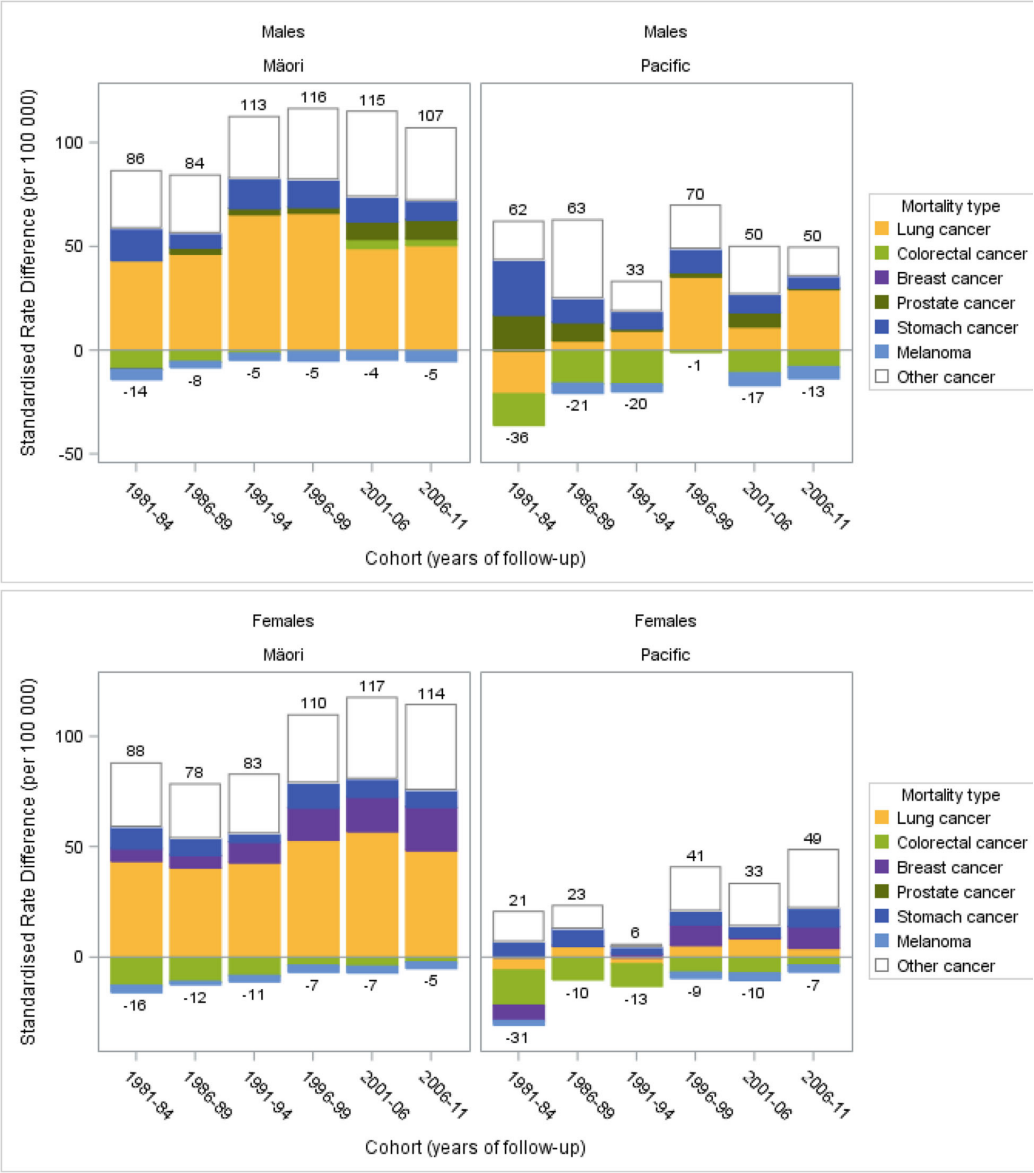
Decomposition of absolute ethnic inequalities in cancer mortality by major contributing cancer types, comparing Māori and Pacific peoples with European/Other in males and females aged 1–74 years in New Zealand. *Note some cancer types are excluded due to small numbers

Table 1 shows ethnic inequalities in cancer mortality increased from 1981 to 2011 particularly in females. For example, in the 2006–11 cohort, the overall mortality rate from cancer was 202.0 per 100 000 Māori females and 92.6 in European/Other, an overall SRD of 109.4 excess deaths per 100 000 (Table 1; and the net height of the 2006–11 bar in Fig. 3, SRD = 114 – 5). From 1981–84 to 2006–11 the cancer mortality in European/Other females decreased significantly from 110.6 to 92.6 per 100 000 population (*p* = 0.01) but the cancer mortality rate for Māori females appeared to have increased (182.8 to 202.0, *p* = 0.09). Subsequently, absolute inequalities (SRD) for Māori females increased from 72.2 to 109.4 deaths per 100 000 (*p* = 0.01) (Table 1; and the net height of the bars in Fig. 3). There was a similar increase in inequalities for Pacific females (SRDs −9.8 to 42.2, *p* = 0.03) and some suggestive increase for Māori males (72.5 to 102.0, *p* = 0.17).

**Table 1:** Trends in the absolute ethnic inequalities in cancer incidence and mortality in Māori and Pacific males and females compared to European/Other, New Zealand census-linked mortality data 1981–2011

Cancer	Cohort (years)	European/Other males		Māori males					Pacific males				
		Rate	Linear trend *p*-value	Rate	Linear trend *p*-value	Rate difference (CI)	Linear trend *p*-value	Rate ratio (CI)	Rate	Linear trend *p*-value	Rate difference (CI)	Linear trend *p*-value	Rate ratio (CI)
Mortality													
Lung	1981–84	44.5	.	87.8	.	43.4 (24.4 to 62.4)	.	1.98 (1.58–2.47)	23.5	.	−21.0 (−39.1– −2.9)	.	0.53 (0.25–1.14)
	2006–11	20.5	<0.01	71.2	0.07	50.7 (42.6–58.8)	0.87	3.47 (3.06–3.94)	49.9	0.30	29.4 (17.9–40.9)	0.07	2.43 (1.92–3.08)
Colorectal	1981–84	22.5	.	13.7	.	−8.8 (−15.5– −2.2)	.	0.61 (0.38–0.98)	7.5	.	−15.0 (−25.6– −4.4)	.	0.33 (0.08–1.35)
	2006–11	17.4	0.05	20.4	0.15	3.1 (−1.1–7.3)	<0.01	1.18 (0.96–1.45)	9.4	0.68	−7.9 (−11.8– −4.0)	0.10	0.54 (0.36–0.81)
Prostate	1981–8	9.1	.	8.8	.	−0.3 (−7.6–7.0)	.	0.97 (0.42–2.21)	26.2	.	17.1 (−14.5–48.6)	.	2.87 (0.85–9.63)
	2006–11	9.3	0.86	18.5	0.01	9.2 (4.8–13.7)	<0.01	1.99 (1.55–2.56)	10.0	0.23	0.7 (−4.7–6.1)	0.32	1.07 (0.62–1.85)
Stomach	1981–84	8.7	.	24.3	.	15.6 (6.2–25.0)	.	2.80 (1.87–4.20)	35.2	.	26.6 (−1.5–54.6)	.	4.07 (1.82–9.10)
	2006–11	3.8	<0.01	13.2	0.15	9.4 (6.1–12.7)	0.57	3.47 (2.62–4.59)	9.7	0.04	5.8 (1.1–10.6)	0.07	2.53 (1.53–4.18)
Melanoma	1981–84	5.6	.	0.8	.	−4.8 (−6.6– −3.1)	.	0.14 (0.02–0.97)	.	.	.	.	.
	2006–11	7.1	.	1.9	.	−5.1 (−6.6– −3.6)	.	0.28 (0.14–0.54)	1.6	.	−5.5 (−7.2– −3.7)	.	0.23 (0.08–0.61)
*All cancers*	1981–84	*147.2*	*.*	*219.7*	*.*	*72.5 (43.5–101.4)*	*.*	*1.49 (1.31–1.71)*	*173.3*	*.*	*26.1 (−31.0–83.1)*	*.*	*1.18 (0.85–1.64)*
	2006–11	*112.2*	*<0.01*	*214.3*	*0.66*	*102.0 (88.1–116.0)*	*0.17*	*1.91 (1.78–2.04)*	*148.4*	*0.20*	*36.2 (17.5–54.9)*	*0.94*	*1.32 (1.17–1.50)*
Incidence													
Lung	1981–86	50.9	.	91.2	.	40.3 (26.7 to 54.0)	.	1.79 (1.54 to 2.09)	56.5	.	5.7 (−26.1 to 37.4)	*.*	1.11 (0.63 to 1.95)
	2006–11	25.6	<0.01	78.9	0.08	53.3 (44.8 to 61.8)	0.69	3.08 (2.74 to 3.47)	49.3	0.15	23.7 (12.3 to 35.2)	0.70	1.93 (1.52 to 2.44)
Colorectal	1981–86	48.8	.	24.5	.	−24.3 (−32.1 to −16.4)	.	0.50 (0.37 to 0.69)	22.3	.	−26.5 (−40.1 to −12.9)	.	0.46 (0.25 to 0.84)
	2006–11	47.4	0.76	42.9	<0.01	−4.5 (−10.7 to 1.8)	<0.01	0.91 (0.78 to 1.05)	29.8	0.94	−17.6 (−26.0 to −9.3)	0.89	0.63 (0.48 to 0.83)
Prostate	1981–86	27.7	.	32.7	.	5.0 (−5.5 to 15.5)	.	1.18 (0.86 to 1.63)	20.7	.	−7.0 (−30.0 to 16.0)	.	0.75 (0.25 to 2.27)
	2006–11	110.1	<0.01	101.0	<0.01	−9.1 (−18.8 to 0.6)	0.11	0.92 (0.83 to 1.01)	88.2	0.01	−21.9 (−36.6 to −7.2)	0.07	0.80 (0.68 to 0.95)
Stomach	1981–86	11.5	.	18.9	.	7.4 (1.8 to 13.1)	.	1.65 (1.21 to 2.24)	50.1	.	38.6 (8.6 to 68.6)	.	4.37 (2.38 to 8.00)
	2006–11	5.5	<0.01	18.7	0.44	13.2 (9.2 to 17.2)	0.53	3.42 (2.69 to 4.34)	15.0	0.13	9.6 (4.2 to 14.9)	0.31	2.75 (1.89 to 3.99)
Melanoma	1981–86	21.1	.	6.4	.	−14.7 (−18.0 to −11.5)	.	0.30 (0.19 to 0.48)	3.6	.	−17.5 (−21.8 to −13.2)	.	0.17 (0.06 to 0.53)
	2006–11	53.3	<0.01	8.0	0.11	−45.3 (−48.3 to −42.2)	<0.01	0.15 (0.11 to 0.20)	3.2	0.78	−50.1 (−53.0 to −47.2)	<0.01	0.06 (0.03 to 0.12)
Liver*	1981–86	1.5	.	8.7	.	7.3 (3.8 to 10.8)	.	5.95 (3.73 to 9.51)	16.3	.	14.8 (7.1 to 22.6)	.	11.12 (6.51 to 19.0)
	2006–11	3.8	0.01	16.5	0.02	12.8 (9.4 to 16.1)	0.04	4.39 (3.46 to 5.58)	19.3	0.82	15.5 (8.9 to 22.1)	0.64	5.12 (3.55 to 7.38)
*First cancers^*	1981–86	*271.2*	*.*	*309.0*	*.*	*37.8 (12.2 to 63.3)*	*.*	*1.14 (1.05 to 1.24)*	*324.8*	*.*	*53.6 (−12.7 to 119.9)*	.	1.20 (0.98 to 1.47)
	2006–11	*401.8*	*<0.01*	*427.1*	*<0.01*	*25.3 (5.6 to 45.0)*	*0.24*	*1.06 (1.01 to 1.11)*	*324.2*	*0.47*	*−77.6 (−105 to −50.1)*	*0.02*	*0.81 (0.74 to 0.88)*
		European/Other females		Māori females					Pacific females				
Cancer	Cohort	Rate	Linear trend *p*-value	Rate	Linear trend *p*-value	Rate difference (CI)	Linear trend *p*-value	Rate ratio (CI)	Rate	Linear trend *p*-value	Rate difference (CI)	Linear trend *p*-value	Rate ratio (CI)
Mortality													
Lung	1981–84	12.1	.	55.7	.	43.7 (29.0–58.3)	.	4.62 (3.49–6.12)	6.1	.	−5.9 (−13.1–1.2)	.	0.51 (0.16–1.62)
	2006–11	16.1	.	64.6	.	48.5 (41.8–55.2)	.	4.01 (3.56–4.52)	20.4	.	4.3 (−1.5–10.0)	.	1.26 (0.95–1.68)
Colorectal	1981–84	19.3	.	6.7	.	−12.6 (−17.4– −7.8)	.	0.35 (0.18–0.68)	3.5	.	−15.8 (−21.4– −10.2)	.	0.18 (0.04–0.85)
	2006–11	13.6	.	11.4	.	−2.2 (−5.4–1.0)	.	0.84 (0.64–1.11)	10.0	.	−3.6 (−7.7–0.5)	.	0.74 (0.49–1.11)
Breast	1981–84	25.6	.	31.3	.	5.7 (−4.0–15.3)	.	1.22 (0.89–1.67)	18.7	.	−6.9 (−21.4–7.5)	.	0.73 (0.34–1.57)
	2006–11	18.2	<0.01	37.8	<0.01	19.6 (14.5–24.6)	<0.01	2.07 (1.80–2.39)	27.9	0.53	9.7 (3.3–16.1)	0.13	1.53 (1.21–1.94)
Stomach	1981–84	3.5	.	13.3	.	9.8 (3.8–15.9)	.	3.80 (2.33–6.18)	10.7	.	7.2 (−5.3–19.7)	.	3.05 (0.94–9.89)
	2006–11	1.6	.	9.4	.	7.8 (5.4–10.3)	.	5.88 (4.26–8.13)	10.1	.	8.5 (4.3–12.6)	.	6.30 (4.00–9.92)
Melanoma	1981–84	3.5	.	0.4	.	−3.1 (−4.2– −2.0)	.	0.12 (0.02–0.86)	1.6	.	−1.9 (−5.2–1.4)	.	0.46 (0.06–3.31)
	2006–11	3.6	.	0.9	.	−2.7 (−3.5– −1.9)	.	0.25 (0.12–0.51)	0.7	.	−2.9 (−4.0– −1.9)	.	0.19 (0.05–0.76)
*All cancers^*	1981–84	*110.6*	*.*	*182.8*	*.*	*72.2 (47.5–97.0)*	*.*	*1.65 (1.44–1.90)*	*100.8*	*.*	*−9.8 (−46.3–26.8)*	*.*	*0.91 (0.63–1.31)*
	2006–11	*92.6*	*<0.01*	*202.0*	*0.09*	*109.4 (97.4–121.4)*	*0.01*	*2.18 (2.05–2.32)*	*134.8*	*0.08*	*42.2 (27.4–57.0)*	*0.03*	*1.46 (1.30–1.63)*
Incidence													
Lung	1981–86	14.9	.	45.7	.	30.9 (21.4 to 40.3)	.	3.08 (2.48 to 3.82)	9.1	.	−5.7 (−12.0 to 0.5)	.	0.61 (0.31 to 1.21)
	2006–11	21.0	0.02	74.3	0.07	53.3 (46.1 to 60.5)	0.09	3.53 (3.17 to 3.94)	23.4	0.03	2.4 (−3.9 to 8.6)	0.07	1.11 (0.85 to 1.46)
Colorectal	1981–86	43.1	.	18.5	.	−24.6 (−30.0 to −19.2)	.	0.43 (0.33 to 0.57)	24.5	.	−18.5 (−33.0 to −4.0)	.	0.57 (0.32 to 1.03)
	2006–11	37.8	0.21	24.6	0.05	−13.2 (−17.5 to −8.8)	<0.01	0.65 (0.55 to 0.77)	20.5	0.75	−17.3 (−23.1 to −11.4)	0.21	0.54 (0.41 to 0.72)
Breast	1981–86	69.4	.	69.1	.	−0.3 (−10.7 to 10.1)	.	1.00 (0.86 to 1.16)	67.5	.	−1.9 (−22.4 to 18.5)	.	0.97 (0.72 to 1.32)
	2006–11	95.8	0.03	140.1	<0.01	44.3 (35.2 to 53.4)	<0.01	1.46 (1.37 to 1.57)	116.0	0.05	20.2 (7.6 to 32.8)	0.40	1.21 (1.09 to 1.35)
Stomach	1981–86	4.9	.	14.7	.	9.8 (5.0 to 14.6)	.	2.98 (2.10 to 4.22)	15.9	.	11.0 (−1.3 to 23.3)	.	3.23 (1.47 to 7.07)
	2006–11	2.3	<0.01	13.6	0.96	11.3 (8.4 to 14.2)	0.32	5.84 (4.47 to 7.64)	8.7	0.14	6.4 (2.6 to 10.2)	0.34	3.73 (2.35 to 5.93)
Melanoma	1981–86	29.2	.	6.2	.	−23.0 (−26.2 to −19.8)	.	0.21 (0.14 to 0.33)	4.1	.	−25.2 (−30.0 to −20.3)	.	0.14 (0.05 to 0.43)
	2006–11	48.8	<0.01	7.7	0.23	−41.1 (−43.8 to −38.4)	<0.01	0.16 (0.12 to 0.20)	2.5	0.54	−46.4 (−48.9 to −43.9)	<0.01	0.05 (0.03 to 0.10)
Liver*	1981–86	0.9	.	6.8	.	5.8 (1.1 to 10.5)	.	7.23 (3.38 to 15.5)	4.6	.	3.7 (−1.5 to 8.9)	.	4.93 (1.53 to 15.9)
	2006–11	1.5	.	4.6	.	3.1 (1.5 to 4.8)	.	3.14 (2.08 to 4.74)	3.8	.	2.4 (−0.2 to 4.9)	.	2.62 (1.32 to 5.22)
Cervix*	1981–86	11.9	.	39.8	.	27.9 (19.6 to 36.2)	.	3.35 (2.67 to 4.19)	25.1	.	13.2 (1.9 to 24.4)	.	2.11 (1.34 to 3.32)
	2006–11	5.0	<0.01	14.2	<0.01	9.2 (6.4 to 12.0)	0.03	2.84 (2.25 to 3.59)	9.7	0.08	4.7 (1.3 to 8.1)	0.40	1.95 (1.35 to 2.81)
Endometrial*	1981–86	11.8	.	18.6	.	6.8 (1.9 to 11.8)	.	1.58 (1.20 to 2.07)	22.9	.	11.1 (−1.7 to 23.9)	.	1.94 (1.10 to 3.41)
	2006–11	12.6	0.25	27.5	0.17	14.9 (10.7 to 19.1)	0.20	2.18 (1.85 to 2.57)	49.8	<0.01	37.2 (29.1 to 45.3)	<0.01	3.95 (3.31 to 4.71)
*First cancers^*	1981–86	*258.0*	*.*	*308.0*	*.*	*50.0 (27.0 to 73.0)*	*.*	*1.19 (1.11 to 1.29)*	*277.3*	*.*	*19.2 (−26.3 to 64.8)*	*.*	*1.07 (0.91 to 1.27)*
	2006–11	*344.7*	*0.01*	*419.9*	*<0.01*	*75.2 (58.5 to 91.9)*	*0.22*	*1.22 (1.17 to 1.27)*	*335.1*	*0.09*	*−9.6 (−32.0 to 12.9)*	*0.33*	*0.97 (0.91 to 1.04)*

Note: *For liver cancer only incidence data were available due to small numbers in mortality data. ^Incidence is first cancer incidence in the follow-up period, mortality is any cancer mortality in the follow-up period. All rates, rate differences and rate ratios are age-standardised using the WHO World Standard Population. Cancers were selected for inclusion if they had a significant incidence rate difference in an ethnic comparison in at least four of the six cohort periods

### Contribution of individual cancers to ethnic inequalities in mortality

The pattern of cancers contributing to absolute inequalities in mortality varied by sex, ethnicity and by time (Table 1). In Māori males for example, the standardised mortality rate from lung cancer was 71.2 per 100 000 compared to only 20.5 per 100 000 in European/Other males in the 2006–11 cohort; excess mortality of 50.7 per 100 000 (Fig. 3). Thus the greatest contributors to Māori male cancer mortality SRDs were lung cancer (50 % of the total), stomach cancer (SRD: 9.4 per 100 000, 9 %), and prostate cancer (SRD: 9.2 per 100 000, 9 %) (Table 1, Fig. 3). For Pacific males, the largest contributors were lung cancer (SRD: 29.4 per 100 000, 81 %) and stomach cancer (SRD: 5.8 per 100 000, 16 %); for Māori females it was lung cancer (48.5 per 100 000, 44 %) and breast cancer (19.6 per 100 000, 18 %); and for Pacific females it was breast cancer (9.7 per 100 000, 23 %) and stomach cancer (8.5 per 100 000, 20 %).

The absolute contribution of individual cancer types to ethnic inequalities in mortality changed significantly from the 1981–84 to the 2006–11 cohort (Table 1, Fig. 3). The SRD from lung cancer mortality peaked in the 1996–99 cohort in Māori males and the 2001–06 cohort in Māori females, and was a significant contributor throughout the study period. Pacific males had increasing rates of excess lung cancer mortality, changing from lower rates than European/Other in 1981–84 (SRD −21.0 per 100 000) to higher in the 2006–11 cohort (SRD 29.4 per 100 000; *p*-value for linear trend = 0.07). In Pacific females there was a similar but lower magnitude transition of SRDs from −5.9 to 4.3 per 100 000.

The breast cancer mortality SRD for Māori females increased from 5.7 per 100 000 in the 1981–84 cohort to 19.6 in 2006–11 (*p* < 0.01). There was also a suggestive increase for Pacific females, from a negative difference (−6.9 per 100 000) in 1981–84 to 9.7 per 100 000 in 2006–11 (*p* = 0.13).

Prostate cancer made an increasing contribution to excess Māori male mortality from a negative SRD (−0.3) in the 1981–84 cohort to an excess of 9.2 per 100 000 in 2006–11 (*p* < 0.01).

Colorectal cancer for Māori males also changed from a negative SRD (−8.8 per 100 000 in 1981–84) to an excess of 3.1 per 100 000 in the 2006–11 cohort (*p* < 0.01). The colorectal cancer mortality SRDs were always negative for Māori females, and Pacific males and females, but trended towards zero over time.

Stomach cancer made a decreasing contribution to excess deaths among Māori and Pacific males from the 1981–84 to 2006–11 cohort, for example the SRD in Pacific males decreased from 26.6 to 5.8 per 100 000 (*p* = 0.07).

### Contribution of incidence to mortality trends

**Fig. 4:**
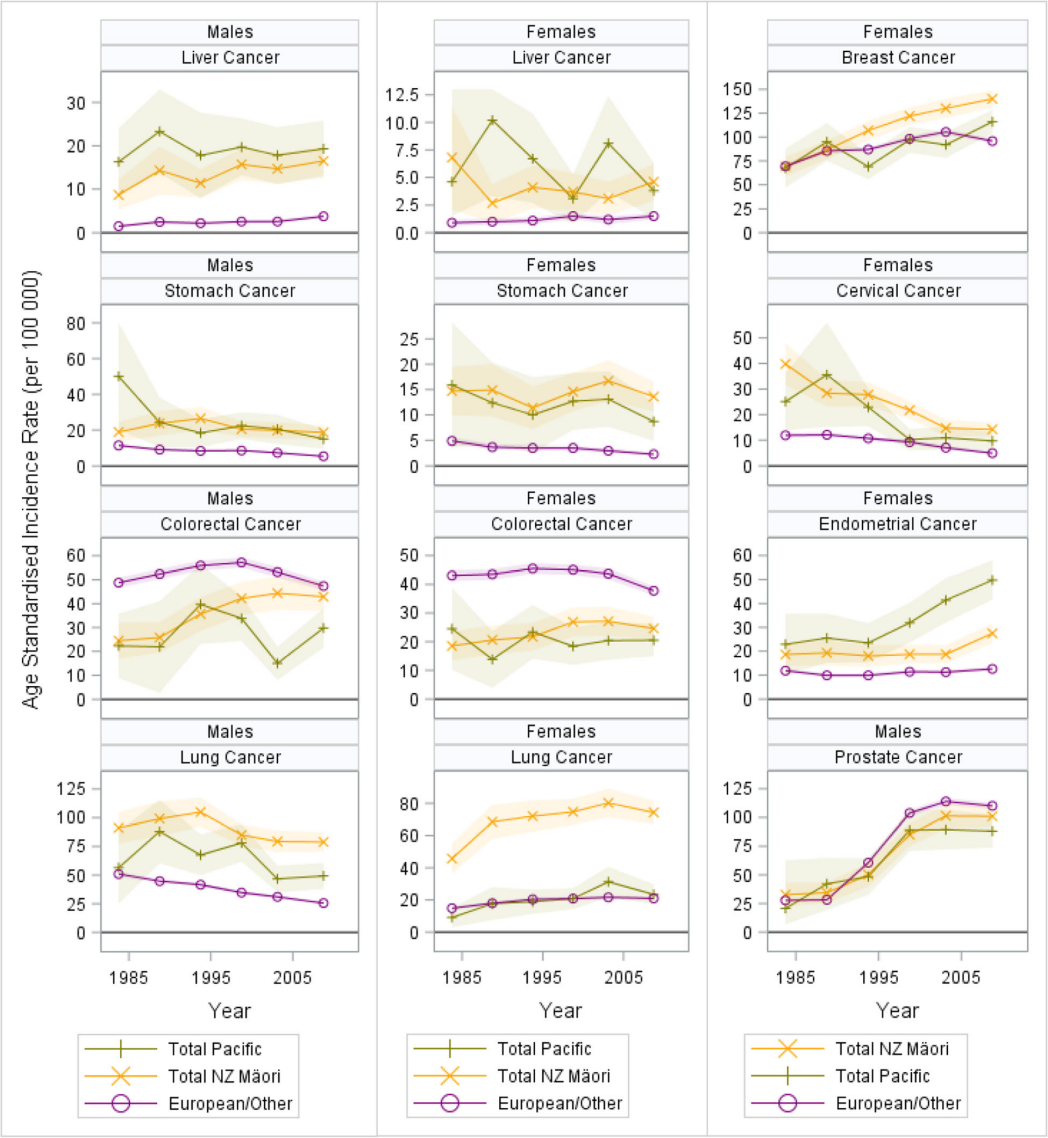
Trends in cancer incidence by ethnic group, males and females aged 1–74 years in New Zealand for six cohorts between 1981 and 2011

**Fig. 5:**
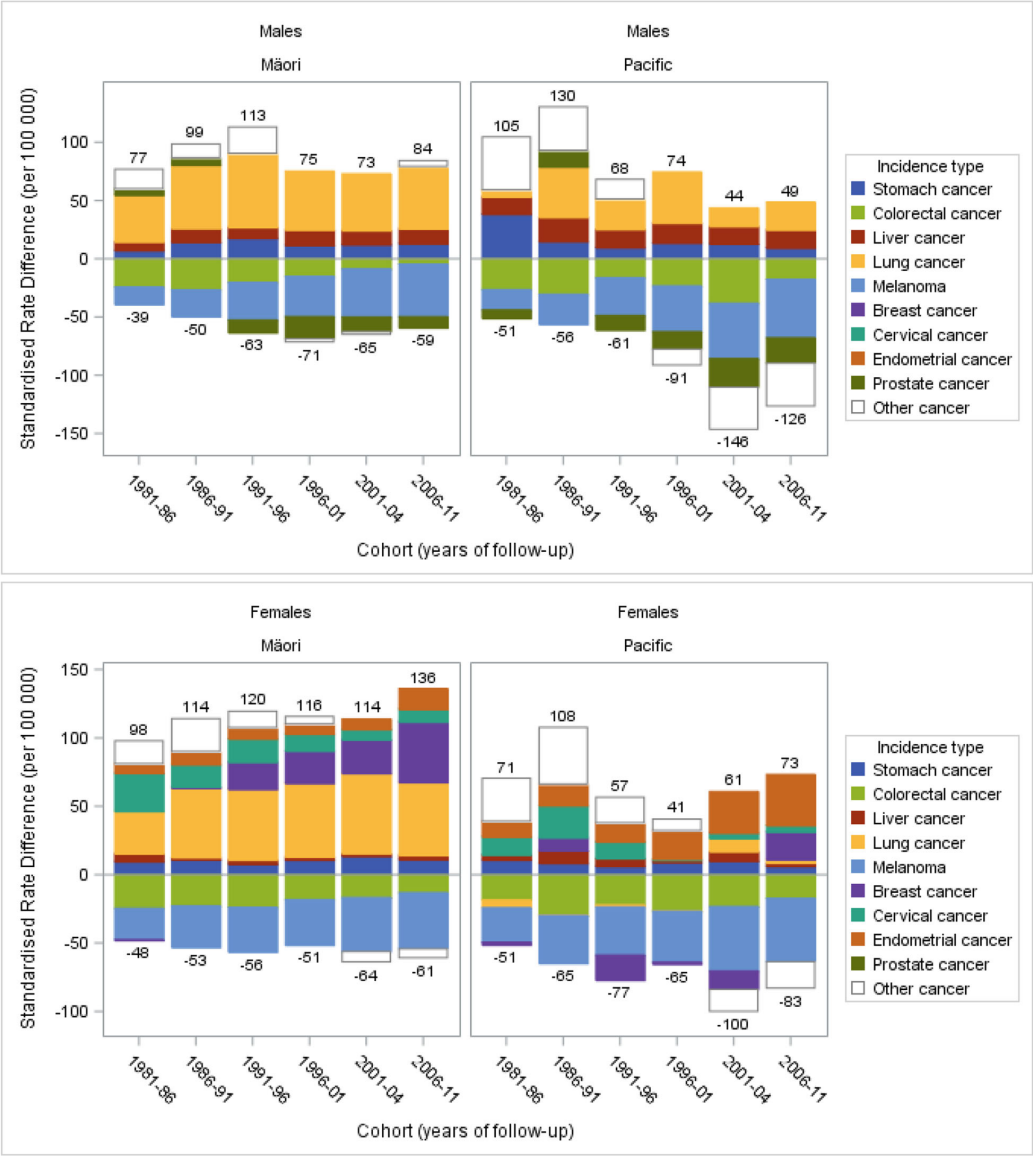
Decomposition of absolute ethnic inequalities in cancer incidence by major contributing cancer types, comparing Māori and Pacific with European/Other in males and females aged 1–74 years in New Zealand

Ethnic inequalities in total cancer incidence varied substantially from the 1981–84 to the 2006–11 cohort. Trends in cancer incidence by ethnicity are shown in Fig. 4, and trends in cancer incidence SRDs in Fig. 5 (with data for endometrial, liver and cervical cancer, not available for mortality trends due to small numbers). The overall cancer incidence gap decreased for: Pacific males (53.6 to −77.6, *p* = 0.02) and probably also so for Māori males (37.8 to 25.3, *p* = 0.24) and Pacific females (19.2 to −9.6, *p* = 0.33). But in Māori females the gap appeared to increase (50.0 to 75.2 per 100 000, *p* = 0.22).

Melanoma contributed to a greater share of incidence inequality trends than mortality trends. Similarly there were diverging trends for prostate cancer mortality and incidence where excess mortality increased (*p* < 0.01) and the incidence gap appeared to decrease (*p* = 0.11).

Melanoma and prostate cancers aside, cancers contributing to the incidence gap were generally similar to the cancers contributing to excess mortality. Breast cancer incidence increased in all ethnic groups but at a faster rate in Māori and Pacific females thus widening the incidence gap (SRD linear trend for both was *p* < 0.01). Protection from colorectal cancer decreased for Māori males (*p* < 0.01) and females (*p* < 0.01) as incidence increased in Māori to levels similar to European/Other. Furthermore, among Pacific females in the 2006–11 cohort, endometrial cancer was the greatest contributor (SRD 37.2 per 100 000) to the incidence gap, and the SRD increased from 11.1 to 37.2 between the 1981–86 and the 2006–11 cohort (*p* < 0.01), with a similar trend in Māori females (SRD from 6.8 to 14.9, *p* = 0.20). In Pacific males liver cancer was the second greatest contributor (15.5 per 100 000) to the incidence gap and its contribution remained stable across time in all groups except for Māori males for whom the gap increased from 7.3 (1981–86) to 12.8 (2006–11) (*p* = 0.04). There were also reductions in the cervical cancer incidence gap in Māori (SRD 27.9 to 9.2, *p* = 0.03) and Pacific females (13.2 to 4.7, *p* = 0.08). Stomach and liver cancer accounted for the greatest relative ethnic inequalities (SRRs) with three- to six-fold greater incidence in Māori and Pacific populations compared to European/Other (2006–11).

## Discussion

This study found that inequalities in cancer mortality increased in Māori males and females, Pacific females and possibly in Pacific males each compared to European/Other. This is similar to evidence of an increased contribution from cancer to socioeconomic inequalities in mortality from Norway [1] and New Zealand [2]. A transition is occurring in which particular diseases contribute to inequalities. From the 1981–84 to the 2006–11 cohort excess cancer mortality (SRDs) significantly increased for breast cancer in Māori females, prostate and colorectal cancers in Māori males, and there was a decreasing trend for stomach cancer in Pacific males. There were significant increases in the contribution (SRDs) of breast cancer (Māori and Pacific females), endometrial cancer (Pacific females) and liver cancer (Māori males) to the ethnic gap in cancer incidence, while the cervical cancer contribution significantly decreased for Māori females. Furthermore, colorectal cancer incidence in Māori males and females increased towards the European/Other incidence thus significantly narrowing the ethnic difference that was previously favouring Māori. In the 2006–11 cohort, lung, breast and stomach cancers made the largest contribution to mortality inequalities in Māori and Pacific peoples, and the largest contributors to the ethnic gap in cancer incidence were lung, breast, stomach, endometrial and liver cancer.

### Incidence drivers: smoking, obesity, alcohol, infection and screening

The trends over time in cancer incidence and mortality, and contribution of individual cancers to excess cancer mortality (for Māori and Pacific) vary by cancer type, and might best be understood by examining changes to the mediating risk factors of tobacco, obesity, alcohol consumption, carcinogenic infectious agents and access to cancer screening (Fig. 1). While this study is of just one high-income country, the implications are potentially relevant to many other countries given the globally shared patterns of epidemiological transitions and (to some extent) common social and ethnic inequalities in the determinants of cancer inequalities.

Smoking-related gaps in lung cancer mortality between Māori and European/Other are starting to diminish, but these gaps continued to increase for Pacific males and females. These patterns align well with ethnic-group specific trends in smoking prevalence (Fig. 1). But even for Māori, the large lung cancer gap means that there is still much more to be gained in terms of reducing ethnic inequalities through enhanced tobacco control.

Absolute ethnic inequalities in cancer increased over our study period for obesity-related breast and endometrial cancers [3], and colorectal, prostate and liver cancers where obesity may play a lesser role. The increasingly higher rates of diabetes, physical inactivity and obesity were calculated to explain 79 % of the endometrial cancer incidence gap for Pacific females compared to European/Other in the 2001–04 cohort [20]. Increasing obesity-related cancer trends are consistent with at least a decade of marked and widening ethnic disparities in obesity (Fig. 1) and the contribution of obesity to increased incidence trends in breast, endometrial, and colorectal cancer in other countries [3]. Similar to our study, in the United States (US) Non-Hispanic Black population (compared to White) there were higher rates of obesity [21], endometrial cancer (7.5 per 100 000 vs 4.0), breast cancer (30.6 vs 21.7), and also colorectal cancer incidence (male 27.7 vs 18.5, female 18.5 vs 13.0) which we did not identify [22]. Greater policy attention is required to constrain the obesogenic environment.

Total alcohol consumption does not differ much between Māori and European/Other and differences by ethnicity have not changed much over time (Fig. 1). European/Other are more likely to have drunk alcohol in the last year but Māori and Pacific (males only) have higher rates of hazardous drinking patterns [23]. However, for Māori and Pacific women there may be some suggestion that alcohol consumption (in the last year) has increased over time to match European/Other and narrowed the gap (Fig. 1). This may have contributed somewhat to the pattern of inequalities observed for breast and colorectal cancer in women, but it does not contribute to explaining the similar patterns in men for colorectal cancer.

There is also more to be gained through addressing ethnic inequalities and the burden of infection-related cancers. Stomach and liver cancer make a large contribution to absolute ethnic inequalities (stomach cancer was the second greatest contributor to ethnic inequalities in mortality for both Māori and Pacific males) and accounted for the greatest relative ethnic inequalities (SRRs). Additional measures to reduce stomach and liver cancer are required, and may include screening and treating for *H. pylori* infection [24]. Inequalities for Māori males in liver cancer incidence increased over our study period, likely relating to trends in chronic hepatitis B infection – which should peak soon with the widespread vaccination against hepatitis B from the late 1980s. The stomach cancer contribution to inequalities somewhat decreased in Māori and Pacific males, paralleling declines elsewhere [22] and declines in *H. pylori* infection [25] which is implicated in 89 % of distal stomach cancer [26].

Trends in cervical and prostate inequalities are potentially related to disparities in access to screening and treatment services. Effective screening programmes with equitable coverage can reduce excess cancer mortality, with the substantial reduction of cervical cancer inequalities seen in New Zealand being an example [27]. Absolute inequalities in cervical cancer incidence have fallen in parallel with recent data on closing inequalities in screening coverage (Fig. 1) and (less importantly) declining smoking prevalence. Prostate cancer mortality inequalities increased and this is likely due to inequitable access to detection and management [16] and perhaps to a lesser extent increased consumption of animal fat, obesity, and physical inactivity [3].

Whilst we do not directly quantify survival inequalities in this paper, Māori have approximately one-third higher deaths rates once diagnosed with cancer (due to comorbidities, later diagnosis and other factors) and there is no evidence that ethnic inequalities in survival have changed over time in New Zealand [28]. This contrasts with the improvement reported for some such cancer survival disparities by ethnicity in the US [29, 30].

### Study strengths and limitations

The linkage of census and cancer and mortality registration data permitted a rigorous analysis of cancer trends by ethnicity over a 30 year period for an entire national population, free of numerator-denominator bias. Study size was very large (87 million person-years of follow-up for cancer incidence) but statistical precision was sometimes limited, for example for Pacific generally and individual cancers such as liver, endometrial and cervical cancer. The ability to examine trends in both incidence and mortality inequalities provided a valuable way to evaluate the consistency of the cancer-specific trends. In addition, mortality data is more robust as it does not rely on diagnostic patterns that reflect clinician effort and thresholds for investigation (for example prostate cancer).

## Conclusions

This study found increases in both the absolute size and percentage of the cancer contribution to excess mortality in Māori compared to European/Other. Some reductions in ethnic inequalities in certain cancers are likely to reflect decades of tobacco control measures and improved cervical screening coverage in New Zealand. Tobacco use remained the greatest contributor to inequalities and there were persistent absolute inequalities in infection-related cancers. The increasing excess incidence and mortality rates in several obesity- and health care access-related cancers provide a sentinel warning of the emerging drivers of ethnic inequalities. Action to further address inequalities in cancer burden needs to be multi-pronged with attention to enhanced control of tobacco, obesity, alcohol, and carcinogenic infectious agents, and focus on addressing access to effective screening and quality health care.

## Additional file

Additional file 1: Figure S1. Hazardous alcohol consumption by ethnicity as measured by the AUDIT tool, score ≥8 for 15+ year olds in the New Zealand Health Survey.
**Figure S2.** Seroprevalence data indicating *H. pylori* prevalence by birth cohort (McDonald et al., 2015) in New Zealand.
**Figure S3.** Mortality rates by ethnicity for all-cause and specific causes of mortality, from national census-linked data in New Zealand males and females 1**–**74 years old 1981**–**2011.
**Figure S4.** Cancer mortality by ethnicity, age standardised, from the national census-linked data in New Zealand males and females 1**–**74 years old 1981**–**2011.
**Figure S5.** Absolute ethnic inequalities (age standardised rate differences) in cancer incidence, from national census-linked data in New Zealand males and females 1**–**74 years old 1981–2011.
**Figure S6.** Absolute ethnic inequalities (age standardised rate differences) in cancer mortality, from national census-linked data in New Zealand males and females 1**–**74 years old 1981**–**2011.
**Figure S7.** Decomposition of absolute ethnic inequalities in cancer mortality (top) and incidence (bottom) by major contributing cancer types, comparing Māori and Pacific peoples with European/Other in males and females aged 1**–**74 years in New Zealand.
**Table S1.** SAS output only showing the statistically significant rate differences used to select cancer incidences for presenting in this paper. (DOCX 840 kb)

## Abbreviations

SRD: Standardised rate differences
SRR: Standardised rate ratio
