# Supplementary material for: Ethnic inequalities in cancer incidence and mortality: census-linked cohort studies with 87 million years of person-time follow-up
Source: BMC Cancer. 2016 Sep 26;16:755. doi: 10.1186/s12885-016-2781-4 (PMC5037611; doi:10.1186/s12885-016-2781-4)
Supplement: Additional file 1: Figure S1. — Hazardous alcohol consumption by ethnicity as measured by the AUDIT tool, score ≥8 for 15+ year olds in the New Zealand Health Survey. Figure S2. Seroprevalence data indicating H. pylori prevalence by birth cohort (McDonald et al., 2015) in New Zealand. Figure S3. Mortality rates by ethnicity for all-cause and specific causes of mortality, from national census-linked data in New Zealand males and females 1–74 years old 1981–2011. Figure S4. Cancer mortality by ethnicity, age standardised, from the national census-linked data in New Zealand males and females 1–74 years old 1981–2011. Figure S5. Absolute ethnic inequalities (age standardised rate differences) in cancer incidence, from national census-linked data in New Zealand males and females 1–74 years old 1981–2011. Figure S6. Absolute ethnic inequalities (age standardised rate differences) in cancer mortality, from national census-linked data in New Zealand males and females 1–74 years old 1981–2011. Figure S7. Decomposition of absolute ethnic inequalities in cancer mortality (top) and incidence (bottom) by major contributing cancer types, comparing Māori and Pacific peoples with European/Other in males and females aged 1–74 years in New Zealand. Table S1. SAS output only showing the statistically significant rate differences used to select cancer incidences for presenting in this paper. (DOCX 840 kb) [file 12885_2016_2781_MOESM1_ESM.docx]

# Additional file 1

**Alcohol and *H. pylori* infection trends**

Figure S1: Hazardous alcohol consumption by ethnicity as measured by the AUDIT tool, score ≥8 for 15+ year olds in the New Zealand Health Survey


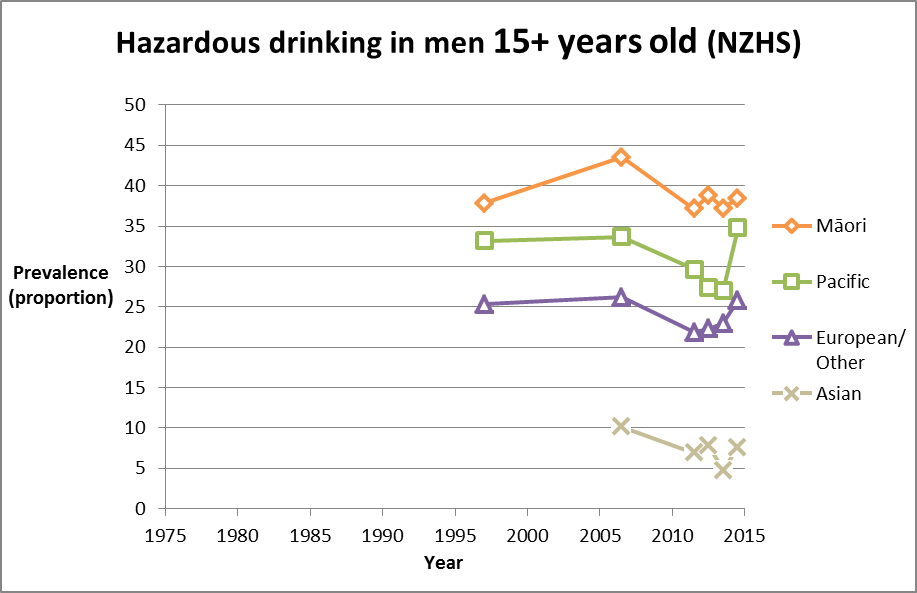

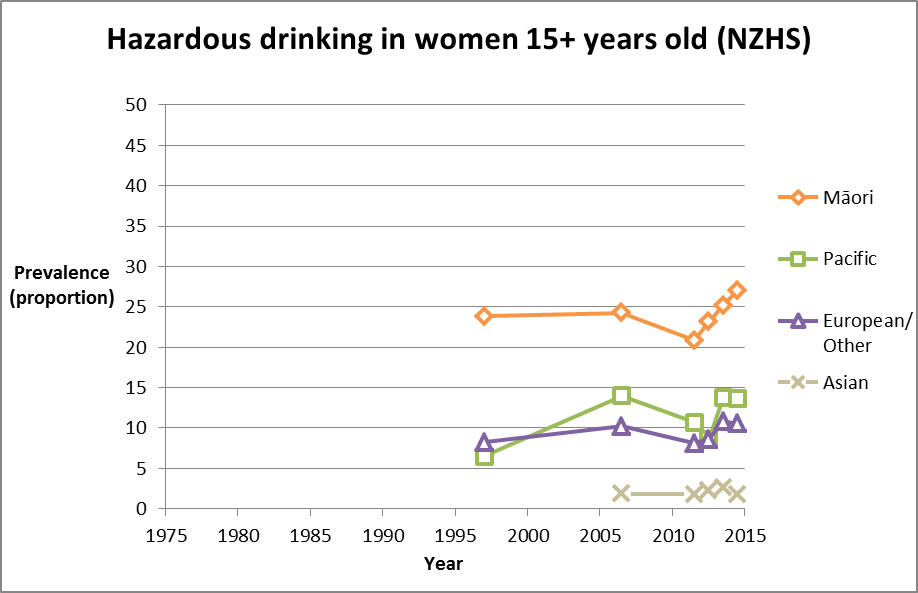


Figure S2: Seroprevalence data indicating *H. pylori* prevalence by birth cohort (McDonald et al., 2015) in New Zealand


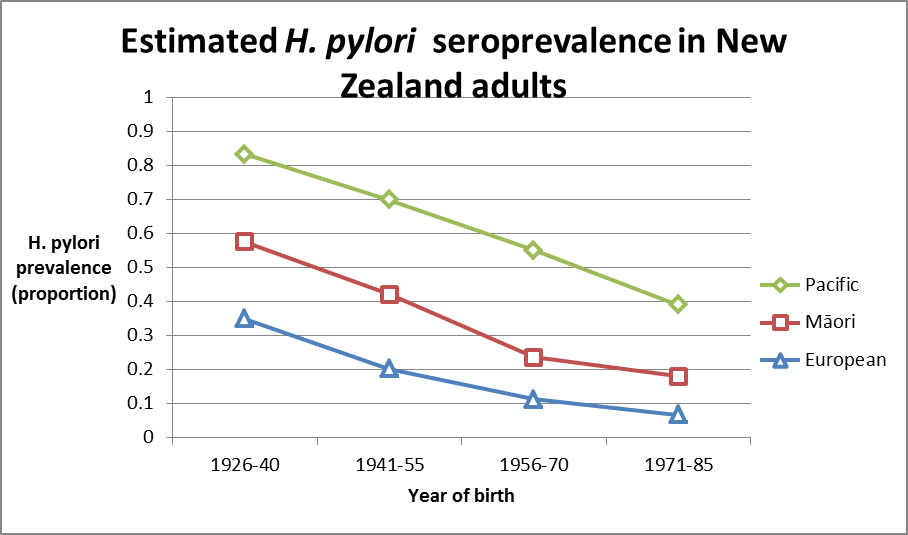


**All-cause mortality rates in the New Zealand population**

Figure S3: Mortality rates by ethnicity for all-cause and specific causes of mortality, from national census-linked data in New Zealand males and females 1-74 years old 1981-2011


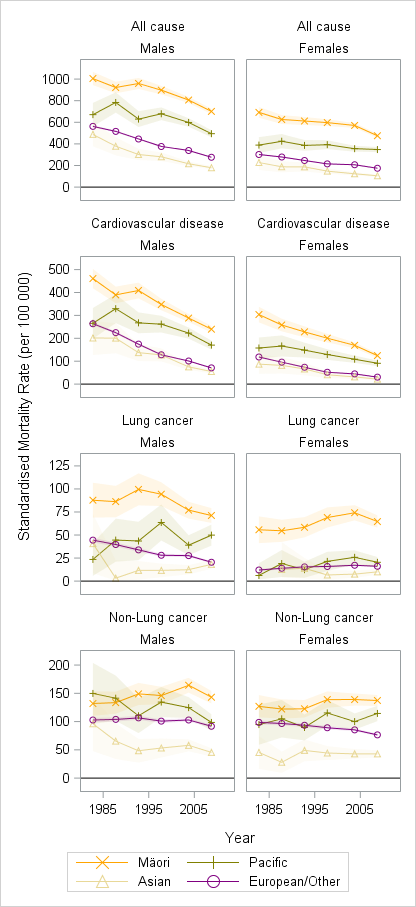

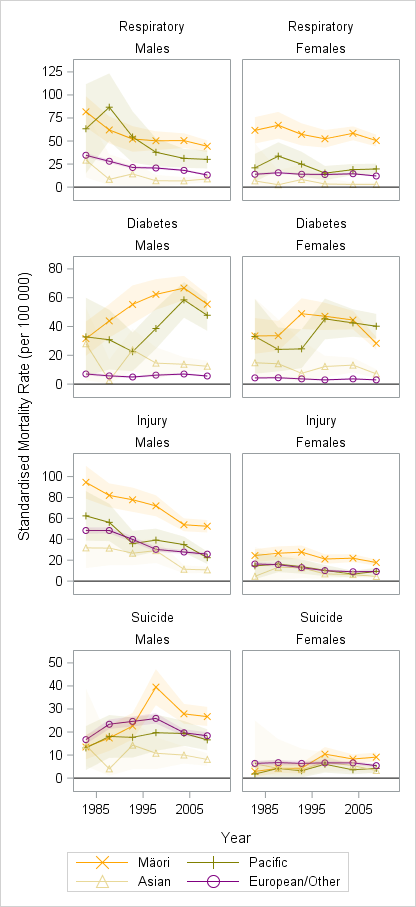


**Cancer incidence and mortality trends by ethnicity**

Figure S4: Cancer mortality by ethnicity, age standardised, from the national census-linked data in New Zealand males and females 1-74 years old 1981-2011

| 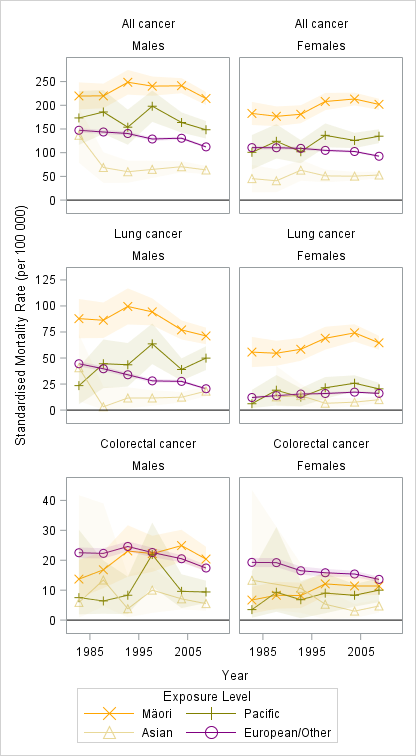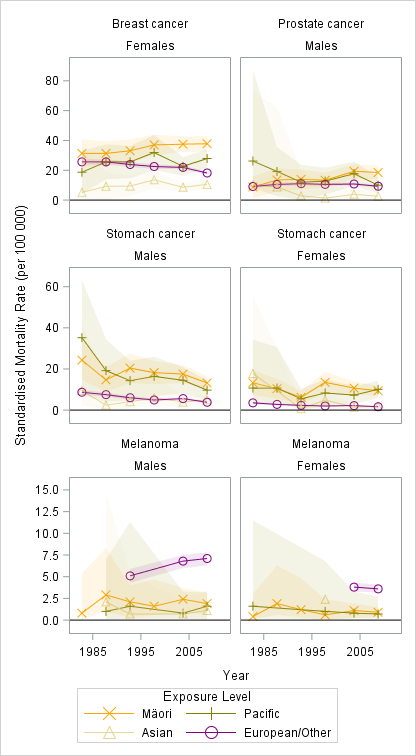 |
| --- |
|  |

**Absolute inequalities in cancer incidence and mortality**

Figure S5: Absolute ethnic inequalities (age standardised rate differences) in cancer incidence, from national census-linked data in New Zealand males and females 1-74 years old 1981-2011


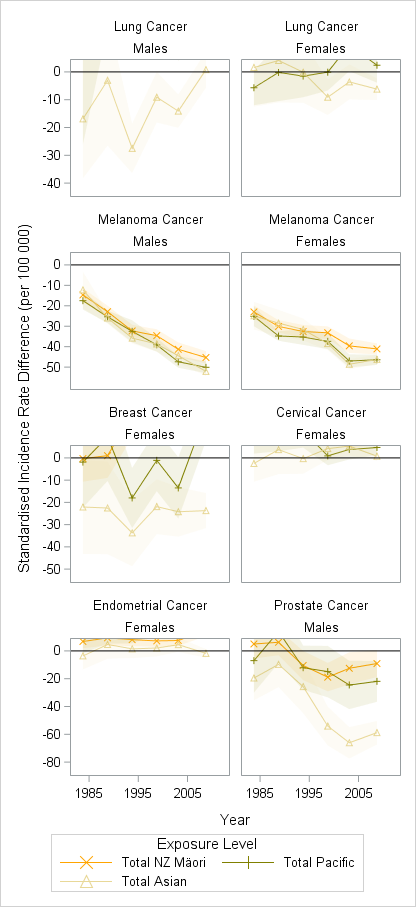

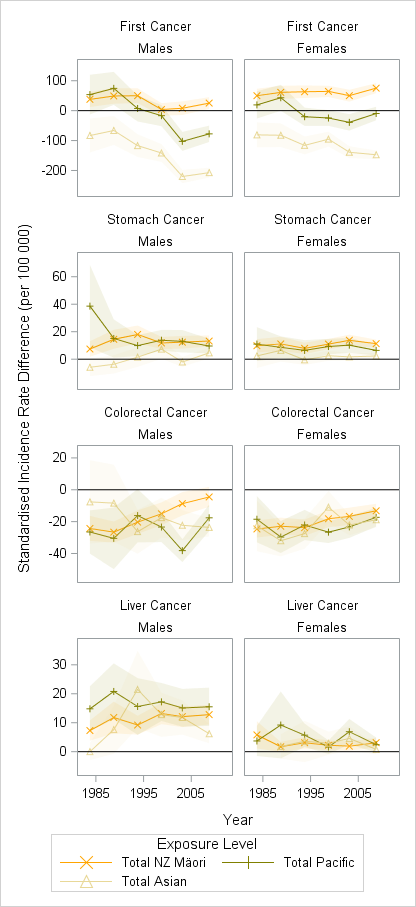

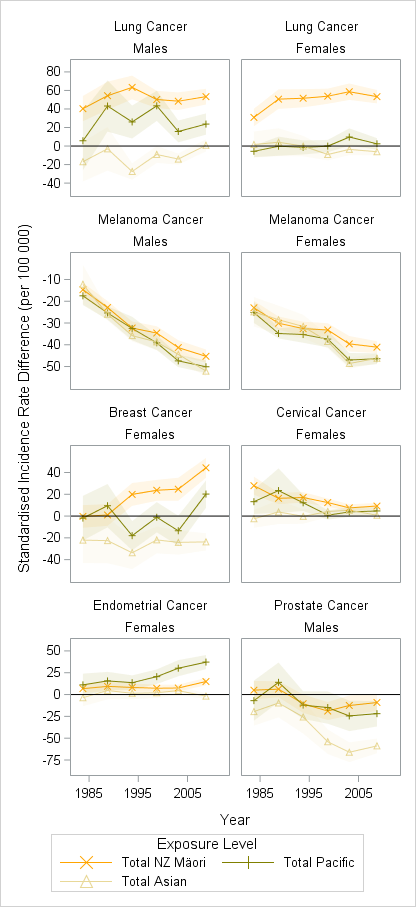


Figure S6: Absolute ethnic inequalities (age standardised rate differences) in cancer mortality, from national census-linked data in New Zealand males and females 1-74 years old 1981-2011


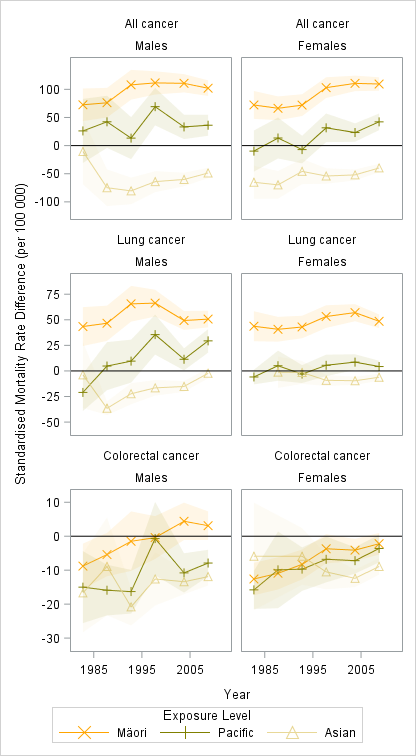

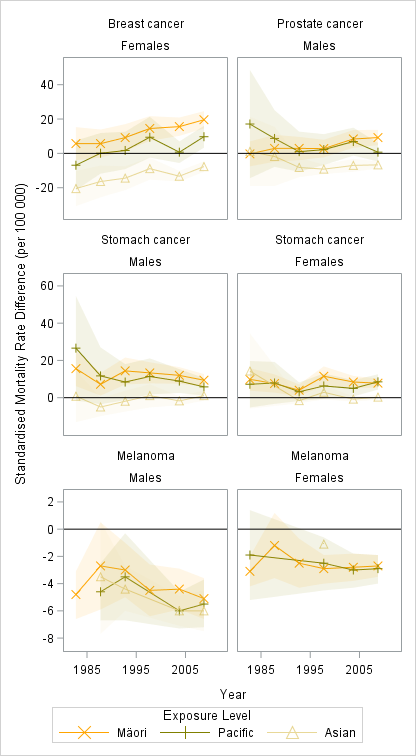


Figure S7: Decomposition of absolute ethnic inequalities in cancer mortality (top) and incidence (bottom) by major contributing cancer types, comparing Māori and Pacific peoples with European/Other in males and females aged 1-74 years in New Zealand

| 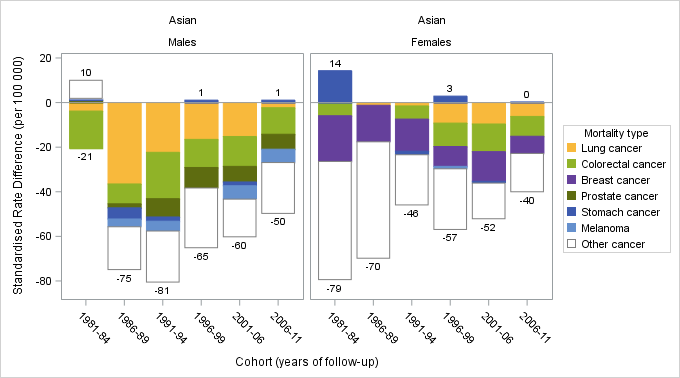 |
| --- |

| 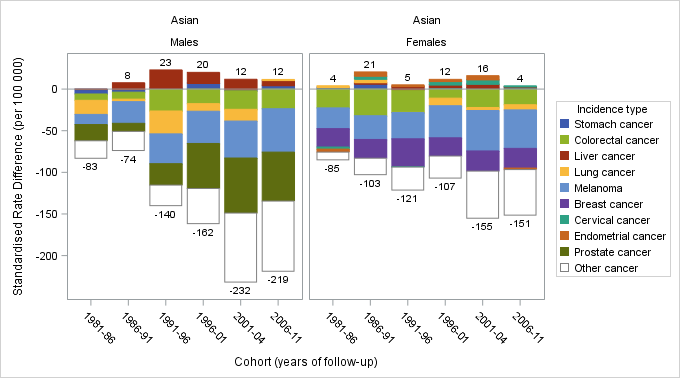 |
| --- |

Table S1: SAS output only showing the statistically significant rate differences used to select cancer incidences for presenting in this paper

|  | | **Males** | | | | | | | | | **Females** | | | | | | | | |
| --- | --- | --- | --- | --- | --- | --- | --- | --- | --- | --- | --- | --- | --- | --- | --- | --- | --- | --- | --- |
|  |  | **Exposure Level** | | | | | | | | | **Exposure Level** | | | | | | | | |
|  |  | **Total NZ Mäori** | | | **Total Pacific** | | | **Total Asian** | | | **Total NZ Mäori** | | | **Total Pacific** | | | **Total Asian** | | |
|  |  | **StdRateDiff** | **SRDCI95Low** | **SRDCI95Upp** | **StdRateDiff** | **SRDCI95Low** | **SRDCI95Upp** | **StdRateDiff** | **SRDCI95Low** | **SRDCI95Upp** | **StdRateDiff** | **SRDCI95Low** | **SRDCI95Upp** | **StdRateDiff** | **SRDCI95Low** | **SRDCI95Upp** | **StdRateDiff** | **SRDCI95Low** | **SRDCI95Upp** |
| **Cancer incidence** | **Year of Census** | . | . | . | 38.60 | 8.60 | 68.60 | . | . | . | . | . | . | . | . | . | . | . | . |
| **Stomach cancer** | **1981-86** |  |  |  |  |  |  |  |  |  |  |  |  |  |  |  |  |  |  |
|  | **1986-91** | 14.50 | 7.60 | 21.40 | 15.10 | 1.10 | 29.00 | . | . | . | 11.10 | 6.00 | 16.30 | . | . | . | . | . | . |
|  | **1991-96** | 18.00 | 11.50 | 24.50 | . | . | . | . | . | . | . | . | . | . | . | . | . | . | . |
|  | **1996-01** | 11.80 | 7.40 | 16.20 | 13.80 | 6.20 | 21.30 | . | . | . | 11.00 | 7.30 | 14.80 | . | . | . | . | . | . |
|  | **2001-4/6** | 12.40 | 7.70 | 17.00 | 13.00 | 4.90 | 21.20 | . | . | . | 13.80 | 9.70 | 17.90 | 10.20 | 4.70 | 15.60 | . | . | . |
|  | **2006-11** | 13.20 | 9.20 | 17.20 | . | . | . | . | . | . | 11.30 | 8.40 | 14.20 | . | . | . | . | . | . |
| **Colorectal cancer** | **1981-86** | -24.30 | -32.10 | -16.40 | -26.50 | -40.10 | -12.90 | . | . | . | -24.60 | -30.00 | -19.20 | -18.50 | -33.00 | -4.00 | -22.60 | -38.60 | -6.70 |
|  | **1986-91** | -26.60 | -33.20 | -19.90 | -30.50 | -49.80 | -11.10 | . | . | . | -22.90 | -28.30 | -17.50 | -29.70 | -39.70 | -19.60 | -32.00 | -41.80 | -22.20 |
|  | **1991-96** | -20.30 | -27.70 | -12.90 | . | . | . | -26.30 | -40.20 | -12.50 | -23.80 | -28.90 | -18.70 | -22.10 | -31.60 | -12.60 | -27.50 | -37.00 | -18.00 |
|  | **1996-01** | -15.10 | -22.20 | -7.90 | -23.40 | -32.90 | -13.80 | -17.50 | -30.00 | -5.10 | -18.20 | -23.50 | -12.90 | -26.60 | -33.40 | -19.80 | -10.90 | -20.90 | -0.80 |
|  | **2001-4/6** | . | . | . | -38.20 | -45.40 | -31.00 | -22.30 | -31.70 | -12.80 | -16.70 | -22.10 | -11.20 | -23.30 | -30.20 | -16.40 | -22.10 | -28.60 | -15.70 |
|  | **2006-11** | . | . | . | -17.60 | -26.00 | -9.30 | -23.60 | -29.30 | -17.80 | -13.20 | -17.50 | -8.80 | -17.30 | -23.10 | -11.40 | -18.80 | -23.60 | -14.00 |
| **Liver cancer** | **1981-86** | . | . | . | 14.80 | 7.10 | 22.60 | . | . | . | . | . | . | . | . | . | . | . | . |
|  | **1986-91** | 11.80 | 6.30 | 17.30 | 20.80 | 11.20 | 30.50 | . | . | . | . | . | . | . | . | . | . | . | . |
|  | **1991-96** | . | . | . | 15.60 | 5.80 | 25.40 | 21.50 | 8.20 | 34.80 | . | . | . | . | . | . | . | . | . |
|  | **1996-01** | 13.20 | 9.50 | 16.90 | 17.20 | 10.60 | 23.70 | 12.80 | 5.10 | 20.40 | . | . | . | . | . | . | . | . | . |
|  | **2001-4/6** | 12.10 | 8.50 | 15.70 | 15.10 | 8.60 | 21.70 | 11.80 | 5.90 | 17.70 | . | . | . | . | . | . | . | . | . |
|  | **2006-11** | 12.80 | 9.40 | 16.10 | 15.50 | 8.90 | 22.10 | . | . | . | . | . | . | . | . | . | . | . | . |
| **Lung cancer** | **1981-86** | 40.30 | 26.70 | 54.00 | . | . | . | . | . | . | 30.90 | 21.40 | 40.30 | . | . | . | . | . | . |
|  | **1986-91** | 54.30 | 39.20 | 69.50 | 43.20 | 15.60 | 70.80 | . | . | . | 50.60 | 40.10 | 61.10 | . | . | . | . | . | . |
|  | **1991-96** | 63.30 | 50.50 | 76.00 | 26.00 | 8.20 | 43.80 | -27.50 | -36.40 | -18.60 | 51.60 | 41.80 | 61.40 | . | . | . | . | . | . |
|  | **1996-01** | 50.20 | 41.00 | 59.50 | 43.40 | 27.10 | 59.70 | . | . | . | 53.80 | 45.60 | 62.00 | . | . | . | . | . | . |
|  | **2001-4/6** | 48.40 | 38.90 | 57.90 | 15.70 | 4.00 | 27.50 | -14.10 | -20.10 | -8.10 | 58.60 | 49.80 | 67.50 | . | . | . | . | . | . |
|  | **2006-11** | 53.30 | 44.80 | 61.80 | 23.70 | 12.30 | 35.20 | . | . | . | 53.30 | 46.10 | 60.50 | . | . | . | . | . | . |
| **Melanoma** | **1981-86** | -14.70 | -18.00 | -11.50 | -17.50 | -21.80 | -13.20 | -12.20 | -21.20 | -3.30 | -23.00 | -26.20 | -19.80 | -25.20 | -30.00 | -20.30 | -24.90 | -31.60 | -18.10 |
|  | **1986-91** | -22.90 | -25.90 | -19.90 | -25.40 | -28.40 | -22.30 | -26.00 | -28.80 | -23.20 | -30.10 | -33.10 | -27.00 | -34.80 | -37.10 | -32.40 | -28.50 | -35.10 | -22.00 |
|  | **1991-96** | -32.30 | -35.30 | -29.30 | -32.50 | -37.90 | -27.10 | -35.80 | -38.50 | -33.10 | -32.60 | -35.80 | -29.40 | -35.30 | -39.10 | -31.50 | -31.60 | -37.00 | -26.20 |
|  | **1996-01** | -34.60 | -37.80 | -31.50 | -39.10 | -42.40 | -35.70 | -38.70 | -42.50 | -34.90 | -33.20 | -36.20 | -30.10 | -37.40 | -41.20 | -33.50 | -38.50 | -41.70 | -35.40 |
|  | **2001-4/6** | -41.30 | -44.80 | -37.90 | -47.40 | -50.30 | -44.60 | -44.40 | -48.00 | -40.90 | -39.60 | -43.10 | -36.00 | -47.00 | -50.20 | -43.80 | -48.60 | -51.10 | -46.00 |
|  | **2006-11** | -45.30 | -48.30 | -42.20 | -50.10 | -53.00 | -47.20 | -52.10 | -54.40 | -49.90 | -41.10 | -43.80 | -38.40 | -46.40 | -48.90 | -43.90 | -46.20 | -48.50 | -43.90 |
| **Breast cancer** | **1981-86** | . | . | . | . | . | . | . | . | . | . | . | . | . | . | . | -22.10 | -43.20 | -1.00 |
|  | **1986-91** | . | . | . | . | . | . | . | . | . | . | . | . | . | . | . | -22.50 | -43.30 | -1.70 |
|  | **1991-96** | . | . | . | . | . | . | . | . | . | 19.90 | 9.30 | 30.40 | -18.00 | -31.50 | -4.50 | -33.70 | -48.70 | -18.70 |
|  | **1996-01** | . | . | . | . | . | . | . | . | . | 23.70 | 14.10 | 33.20 | . | . | . | -21.90 | -34.30 | -9.60 |
|  | **2001-4/6** | . | . | . | . | . | . | . | . | . | 24.70 | 14.10 | 35.20 | . | . | . | -24.20 | -35.40 | -13.10 |
|  | **2006-11** | . | . | . | . | . | . | . | . | . | 44.30 | 35.20 | 53.40 | 20.20 | 7.60 | 32.80 | -23.70 | -31.60 | -15.80 |
| **Cervical cancer** | **1981-86** | . | . | . | . | . | . | . | . | . | 27.90 | 19.60 | 36.20 | 13.20 | 1.90 | 24.40 | . | . | . |
|  | **1986-91** | . | . | . | . | . | . | . | . | . | 16.30 | 11.00 | 21.50 | 23.40 | 3.00 | 43.80 | . | . | . |
|  | **1991-96** | . | . | . | . | . | . | . | . | . | 17.10 | 12.30 | 22.00 | 12.20 | 4.60 | 19.70 | . | . | . |
|  | **1996-01** | . | . | . | . | . | . | . | . | . | 12.40 | 8.80 | 16.00 | . | . | . | . | . | . |
| **Endometrial cancer** | **1986-91** | . | . | . | . | . | . | . | . | . | . | . | . | 15.60 | 5.20 | 26.00 | . | . | . |
|  | **1991-96** | . | . | . | . | . | . | . | . | . | . | . | . | 13.60 | 5.40 | 21.90 | . | . | . |
|  | **1996-01** | . | . | . | . | . | . | . | . | . | . | . | . | 20.50 | 12.10 | 28.90 | . | . | . |
|  | **2001-4/6** | . | . | . | . | . | . | . | . | . | . | . | . | 30.20 | 20.90 | 39.40 | . | . | . |
|  | **2006-11** | . | . | . | . | . | . | . | . | . | 14.90 | 10.70 | 19.10 | 37.20 | 29.10 | 45.30 | . | . | . |
| **Prostate cancer** | **1981-86** | . | . | . | . | . | . | -19.50 | -35.60 | -3.40 | . | . | . | . | . | . | . | . | . |
|  | **1991-96** | -10.50 | -21.00 | 0.00 | . | . | . | -25.70 | -44.80 | -6.50 | . | . | . | . | . | . | . | . | . |
|  | **1996-01** | -18.90 | -29.10 | -8.60 | . | . | . | -54.10 | -68.10 | -40.10 | . | . | . | . | . | . | . | . | . |
|  | **2001-4/6** | -12.50 | -24.10 | -1.00 | -24.50 | -41.90 | -7.10 | -66.10 | -77.40 | -54.80 | . | . | . | . | . | . | . | . | . |
|  | **2006-11** | . | . | . | -21.90 | -36.60 | -7.20 | -58.80 | -67.30 | -50.30 | . | . | . | . | . | . | . | . | . |
| **Bladder cancer** | **1996-01** | . | . | . | -10.70 | -14.20 | -7.20 | . | . | . | . | . | . | . | . | . | . | . | . |
|  | **2001-4/6** | . | . | . | -11.10 | -15.80 | -6.40 | . | . | . | . | . | . | . | . | . | . | . | . |
| **Thyroid cancer** | **1981-86** | . | . | . | . | . | . | . | . | . | . | . | . | 16.00 | 5.80 | 26.20 | . | . | . |
|  | **1986-91** | . | . | . | . | . | . | . | . | . | . | . | . | 11.20 | 2.40 | 20.00 | . | . | . |
|  | **1996-01** | . | . | . | . | . | . | . | . | . | . | . | . | 13.30 | 7.90 | 18.60 | 10.40 | 4.20 | 16.60 |
